# Phylogenomic analysis of 20S proteasome gene family reveals stress-responsive patterns in rapeseed (*Brassica napus* L.)

**DOI:** 10.3389/fpls.2022.1037206

**Published:** 2022-10-31

**Authors:** Vivek Kumar, Hemant Sharma, Lalita Saini, Archasvi Tyagi, Pooja Jain, Yogita Singh, Priyanka Balyan, Sachin Kumar, Sofora Jan, Reyazul Rouf Mir, Ivica Djalovic, Krishna Pal Singh, Upendra Kumar, Vijai Malik

**Affiliations:** ^1^ Department of Botany, Chaudhary Charan Singh University, Meerut, UP, India; ^2^ Department of Genetics and Plant Breeding, Chaudhary Charan Singh University, Meerut, UP, India; ^3^ Department of Molecular Biology & Biotechnology, College of Biotechnology, Chaudhary Charan Singh (CCS) Haryana Agricultural University, Hisar, India; ^4^ Department of Botany, Deva Nagri Post Graduate (PG) College, Chaudhary Charan Singh (CCS) University, Meerut, India; ^5^ Division of Genetics and Plant Breeding, Faculty of Agriculture, Sher-e-Kashmir University of Agricultural Sciences & Technology (SKUAST)-Kashmir, Wadura, India; ^6^ Institute of Field and Vegetable Crops, National Institute of the Republic of Serbia, Maxim Gorki, Novi Sad, Serbia; ^7^ Biophysics Unit, College of Basic Sciences & Humanities, Govind Ballabh (GB) Pant University of Agriculture & Technology, Pantnagar, India; ^8^ Vice-Chancellor’s Secretariat, Mahatma Jyotiba Phule Rohilkhand University, Bareilly, India

**Keywords:** *Brassica napus*, 20S proteasome, phylogenomics, digital expression, environmental stress

## Abstract

The core particle represents the catalytic portions of the 26S proteasomal complex. The genes encoding α- and β-subunits play a crucial role in protecting plants against various environmental stresses by controlling the quality of newly produced proteins. The 20S proteasome gene family has already been reported in model plants such as Arabidopsis and rice; however, they have not been studied in oilseed crops such as rapeseed (*Brassica napus* L.). In the present study, we identified 20S proteasome genes for α- (PA) and β-subunits (PB) in *B. napus* through systematically performed gene structure analysis, chromosomal location, conserved motif, phylogenetic relationship, and expression patterns. A total of 82 genes, comprising 35 *BnPA* and 47 *BnPB* of the 20S proteasome, were revealed in the *B. napus* genome. These genes were distributed on all 20 chromosomes of *B. napus* and most of these genes were duplicated on homoeologous chromosomes. The *BnPA* (α1-7) and *BnPB* (β1-7) genes were phylogenetically placed into seven clades. The pattern of expression of all the *BnPA* and *BnPB* genes was also studied using RNA-seq datasets under biotic and abiotic stress conditions. Out of 82 *BnPA/PB* genes, three exhibited high expression under abiotic stresses, whereas two genes were overexpressed in response to biotic stresses at both the seedling and flowering stages. Moreover, an additional eighteen genes were expressed under normal conditions. Overall, the current findings developed our understanding of the organization of the 20S proteasome genes in *B. napus*, and provided specific *BnPA/PB* genes for further functional research in response to abiotic and biotic stresses.

## 1 Introduction

Rapeseed (*Brassica napus* L.; 2n=38; AACC genome) is an allotetraploid species that is derived through interspecific hybridization of turnip rape (*B. rapa*; 2n=20; AA) with cabbage (*B. oleracea*; 2n=18; CC) (Peng et al., 1999; Chalhoub et al., 2014). Globally, *B. napus* is one of the major oil crops and ranks third in vegetable oil production. It is commonly used as edible oil (Maršalkienė et al., 2009; Lozano-Baena et al., 2015), condiments (Gilbert et al., 2012, Wiersema and Leon, 2016) and fodder (Cartea et al., 2005) in many parts of the world. In addition, canola oil has medicinal properties including a diuretic (Lust, 1983; Grieve, 1984), analgesic and anticancer activities (Duke, 1983). The crop is adapted to the temperate regions of the world and is therefore, very sensitive to a series of environmental threats such as fungal/bacterial diseases, and cold, heat, and drought stresses. These stresses limit the growth and development at different stages during the crop cycle (Kutcher et al., 2010). The presence of high-quality genome sequences and bioinformatics toolkits may help to identify various genes or gene families to elucidate their functional relevance to environmental stress factors to breed climate-smart cultivars.

The ubiquitin-mediated proteolysis system (UPS) also known as the ubiquitin-proteasome pathway (UPP), regulates the degradation of many proteins in eukaryotic cells (Vierstra, 2009). The UPS consists of two distinct, consecutive steps: ubiquitylation (identification of a substrate) and proteasomal degradation (elimination of the ubiquitinated protein) (Kleiger and Mayor, 2014; Sharma et al., 2016). Briefly, ubiquitination is a complicated process that usually necessitates the use of three enzymes: E1, E2, and E3. Ubiquitin is covalently attached to substrate proteins catalyzed by a cascade of enzymes consisting of ubiquitin activator (E1), conjugase (E2), and ligase (E3). Ubiquitin is commonly conjugated to an internal lysine (Lys) residue but can also be conjugated to the free amino terminus of the substrate *via* its carboxy-terminal glycine. A polyubiquitin chain generated by multiple rounds of ubiquitylation can serve as a signal for degradation by the 26S proteasome machinery, a multiprotein complex consisting of a 20S core particle and 19S regulatory particles (Kleiger and Mayor, 2014). The proteasome has two main origins: the primitive form seen in *Thermoplasma acidophilum* (Archaea bacteria) and the evolutionarily improved form found in yeast, plants, and mammals. Numerous investigations in eukaryotes have been conducted to understand the dynamics of the proteasomal complex during the last decade (Dantuma and Bott, 2014; Stone, 2019).

The genetics of 20S proteasome core particles in plant species, including Arabidopsis (Yang et al., 2004), rice (Fu et al., 1998) and wheat (Sharma et al., 2022), have been well studied. Morphologically, the 20S core particle is barrel-shaped, and is composed of 28 nonidentical subunits arranged in 4 axially stacked rings. The rings at the two ends are identically formed by 7α subunits, and the rings in the middle are identically formed by 7β subunits. This gives rise to the 20S core particle having a symmetric configuration of α1-7/β1-7/β1-7/α1-7 (Chen and Hochstrasser, 1996; Wolf and Hilt, 2004). The β-subunits β1, β2, and β5 have proteolytic properties for different substrates. The protein enters the 20S core particle through α-subunits present at the top, and after processing this protein comes out through the α-subunits located at the bottom of the core particle (Chen and Hochstrasser, 1996). In Arabidopsis and many eukaryotes, there are 7α- (α1-7) and 7β-subunits (β1-7). Based on nomenclature, these 7 α- and 7 β-subunits can be represented as proteasome alpha A-G and proteasome beta A-G (i.e., PAA-PAG and PBA-PBG) respectively. There are 23 genes encoding 12 α- and 11 β-subunits in Arabidopsis (Fu et al., 1998). Of these subunits, one gene encodes four subunits (1α and 3β) whereas two genes encode the rest of the subunits. In common wheat (*Triticum aestivum*), 67 members of the 20S proteasome α (*TaPA*) and β (*TaPB*) gene family have recently been discovered (Sharma et al., 2022). These 67 *TaPA* and *TaPB* genes were distributed in all 21 chromosomes of wheat and some of them were found to be involved in heat/drought stress tolerance.

Phylogenomics uses genome-wide data to infer the evolution of genes, genomes, and the tree of life.  (Eisen, 1998). The term conventional phylogenetics is based upon the study of a few genes or morphology whereas phylogenomics is an update of the term phylogenetics and concentrates on genome-level analysis (DeSalle et al., 2020). According to the principle of phylogenomics, orthologous sequences conserve more protein function than paralogous sequences. The most commonly used data in phylogenomics are sequence data including whole genome nucleotide sequences, orthologous genomic blocks, core genomes, core coding genomes, exons, introns, and other conserved biological molecules. The present study involves a phylogenomic analysis of the *B. napus* 20S proteasome gene family and its putative role in biotic and abiotic stresses.

## 2 Materials and methods

### 2.1 Analysis of gene sequence

#### 2.1.1 Identification and retrieval of 20S proteasome gene sequences

Since 20S proteasome family genes have already been characterized in *Arabidopsis thaliana* and rice (*Oryza sativa*), the DNA and protein sequences of *AtPA/PB* and *OsPA/PB* were used as a reference to detect the true orthologs in the *B. napus* genome. Criteria for the identification of true orthologs that were developed (Dhaliwal et al., 2014) were strictly followed in the present analysis. The coding DNA sequences of 20S proteasome family genes related to *A. thaliana* and rice were retrieved from the Ensembl Plants database (https://plants.ensembl.org/index.html). Consequently, coding sequences of 24 genes of *A. thaliana* and 23 genes of rice were subjected to tBLASTx and BlastP searches against the *B. napus* genome assembly available at the Ensembl Plants database (https://plants.ensembl.org/Brassica_napus/Info/Index?db=core).

#### 2.1.2 Structural features of the 20S proteasome gene family

The tools of Ensembl Plants (https://plants.ensembl.org/index.html), Multiple Em for Motif Elicitation Suite Version 5.4.1 (MEME v5.4.1; https://meme-suite.org/meme/tools/meme) and TB tools (https://github.com/CJ-Chen/TBtools/releases) were employed for gene structure analysis. RepeatMaskerv4.0.9 (https://www.repeatmasker.org/cgi-bin/WEBRepeatMasker) and BatchPrimer3 v1.0 (http://probes.pw.usda.gov/batchprimer3) were used to identify transposable elements (TEs) and microsatellites or simple sequence repeats (SSRs) in gene sequences, respectively. The Plant Care database was used to search for the presence of cis-acting regulatory elements in gene sequences, 1500 base pairs (bp) upstream of the promoter region (http://bioinformatics.psb.ugent.be/webtools/plantcare/html/). The plant small RNA target analysis server psRNATarget (https://www.zhaolab.org/psRNATarget/) was used to search for putative microRNAs and their targets in the BnPA/BnPB genes of *B. napus*. Here, 0-3 e-value was used (Dai and Zhao, 2011; Dai et al., 2018). All the abovementioned analyses were performed with default parameters.

#### 2.1.3 Gene duplication and synteny analysis

The gene tree pipeline available in the Ensembl Plants database (Vilella et al., 2009; Bolser et al., 2015) was utilized to infer evolutionary relationships among proteasome genes (*BnPA* and *BnPB*) with the help of a gene identifier. A phylogenetic tree of homologous genes across the genome of *B. napus* and Arabidopsis was constructed using the Plant Compara option. This gene tree was further utilized to identify duplication and speciation events. Synteny and collinearity between chromosomes of *B. napus* and Arabidopsis were determined covering the stretch of 25 genes. For this purpose, the genome browser biotool GENOMICUSv49.01 (https://www.genomicus.bio.ens.psl.eu/genomicus-plants-49.01/cgi-bin/search.pl) was used.

### 2.2 Analysis of protein sequence

#### 2.2.1 Structure, conserved motifs and physio-chemical properties of protein sequences

The CD-search program of the conserved domain database (CDD) at NCBI was used to identify domain features in BnPA/BnPB protein sequences. The physiochemical properties, including amino acid composition, molecular weight, theoretical PI, number of positively/negatively charged residues, instability index, aliphatic index, and grand average of hydropathy (GRAVY) were computed using ExPASy’s ProtParam tool (https://web.expasy.org/protparam/). The network protein sequence (NPS) analysis was performed using a self-optimized prediction method with an alignment (SOPMA) tool (https://npsa-prabi.ibcp.fr/cgi-bin/npsa_automat.pl?page=/NPSA/npsa_sopma.html). Motifs in protein sequences were searched using the MEME suite (https://meme-suite.org/meme/tools/meme). Annotation and visualization of identified motifs were performed using InterPro Scan (https://www.ebi.ac.uk/interpro/search/sequence/) and TB tool, respectively.

#### 2.2.2 Computational analysis, structure and validation of predicted proteins

Homology modeling (HM) was applied to deduce the 3D structure of the predicted proteins. For HM, PSI-BLAST was performed against two databases, the Swiss Model template library (https://swissmodel.expasy.org/), and the protein data bank (http://www.rcsb.org/pdb/home/home) with 100 iterations. A structure analysis and validation server (SAVES; https://saves.mbi.ucla.edu/) was used to verify the predicted 3D structures of *BnPA* and *BnPB* proteins. The relative proportion of amino acids that appear in the favored region was found with the help of the PROCHECK option of SAVES v6.0 (Laskowski et al., 1993). VERIFY 3D was utilized to determine the compatibility of the atomic model (3D) with its amino acid sequence (1D) (Eisenberg et al., 1997). The ERRAT program was used to verify the protein structures through patterns of nonbonded interactions among C, N, and O atoms (Colovos and Yeates, 1993).

#### 2.2.3 Functional annotation and superimposition of 3D structures

The Flexible structure AlignmenT by Chaining Aligned Fragment pairs allowing Twists (FATCAT) server (https://fatcat.godziklab.org/fatcat/fatcat_pair.html) was used to compare the 3D structure of the proteins encoded by various genes belonging to *A. thaliana* with that of predicted *B. napus* proteins by aligning the representative structure. The similarity of 3D structures was measured by the root mean square deviation (RMSD) value of the Cα atoms.

### 2.3 Phylogenetic analysis

The MultAlin program (http://multalin.toulouse.inra.fr/multalin/) was used for multiple sequence alignments to determine conserved amino acid residues in *B. napus*, Arabidopsis and rice. A mutual information server to infer coevolution (MISTIC; http://mistic.leloir.org.ar/results.php?jobid=202112021211022296) was employed to determine the mutual information (MI) between two amino acid positions in multiple sequence alignment (MSA). The identical amino acid between two positions (homologous protein), called mutual information (MI), was predicted to correlate and compensate for mutations, and was used to identify coevolving residues (Sharma et al., 2022). The amino acid sequences of proteins were utilized for phylogenetic analysis by molecular evolutionary genetics analysis version 6.0 (MEGA v6.0) software using the neighbor-joining method (Tamura et al., 2011). The Newick format tree prepared in MEGA was visualized in iTOL (https://itol.embl.de/).

### 2.4 *In silico* expression profile of 20S proteasome genes

Digital expression of *BnPA* and *BnPB* genes was analyzed using transcriptomic data available at *the Brassica expression database* (https://brassica.biodb.org/). Expression was studied in FPKM values using six different tissues (seed, cotyledon, hypocotyls, radical, root, & leaf) belonging to the germination and seedling stages under normal conditions. Under both biotic and abiotic stress conditions, expression was observed at the seedling and flowering stages. Under both normal and stress conditions, the expression pattern of the selected genes was studied.

## 3 Result

### 3.1 Analysis of gene sequence

#### 3.1.1 Identification of genes for the 20S proteasome in *B. napus*

**Figure 1:**
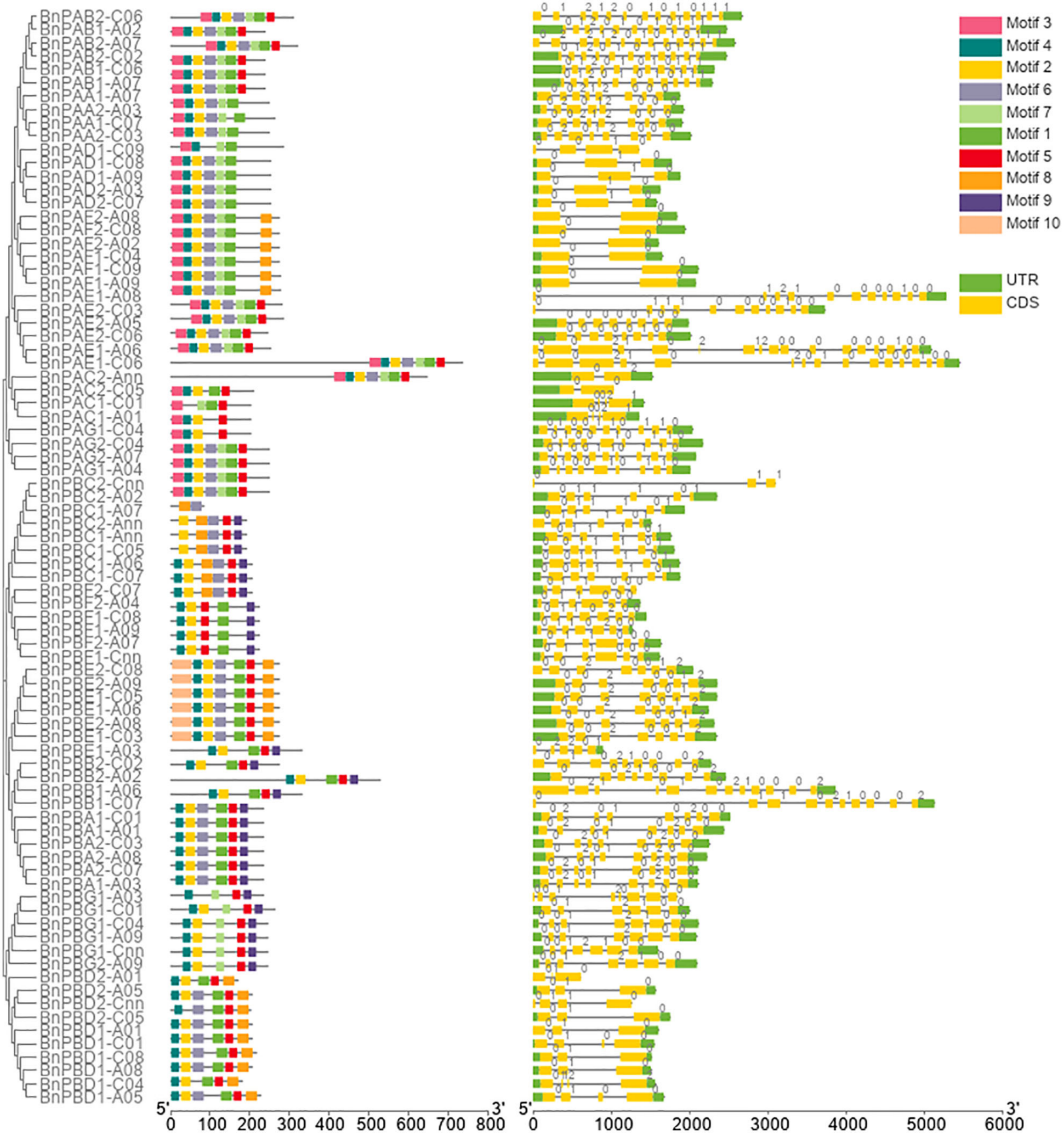
Structure of *BnPA* and *BnPB* genes of *Brassica napus* showing the distribution of exons (yellow solid bars), introns (black lines), upstream/downstream regions (solid green bars), and intron phases marked as 0, 1 and 2. The figure also represents the conserved motifs identified in BnPA and BnPB proteins.

Out of 92 20S proteasome genes initially identified in the *B. napus* genome, 10 genes were incomplete; therefore, these genes were removed from further analysis. The remaining 82 full-length genes involving 35 *BnPA* and 47 *BnPB* of the 20S proteasome family were further characterized in the present study. Comprehensive information on the full-length gene and coding sequences of *B. napus* α-subunits (*BnPAA-BnPAG*) and β-subunits (*BnPBA-BnPBG*) and their comparison with corresponding *A. thaliana* and rice genes are provided in **Supplementary Table S1**. The name of all 82 *B*. *napus* genes of the 20S proteasome family was designated according to the corresponding genes reported earlier for Arabidopsis, rice and wheat (Fu et al., 1998; Sassa et al., 2000; Sharma et al., 2022). The sizes of the *BnPA* and *BnPB* genes varied from 1039-5449 bp and 618-5132 bp, respectively. The cDNA sequence of *BnPA* genes ranged from 852 to 2355 bp and that of *BnPB* genes ranged from 249 to 1818 bp. Furthermore, variations in coding DNA sequence (CDS) were also observed individually in *BnPA* (606-2199 bp) and *BnPB* (249-1578 bp) genes (**Supplementary Table S1**). The intron-exon features of *B. napus* 20S proteasome genes were studied to gain an improved understanding of their structural patterns. The number of exons and introns in *BnPA* genes varied from 2-17 and 1-16, respectively. All 35 *BnPA* genes had introns. The number of exons and introns in *BnPB* genes varied from 3-12 and 2-11, respectively (**Supplementary Table S2**). The intron phases were phase 0 (56.75%), phase 1 (30.45%) and phase 2 (12.79%) (**Figure 1**).

#### 3.1.2 Assignment of chromosome and gene duplication

**Figure 2:**
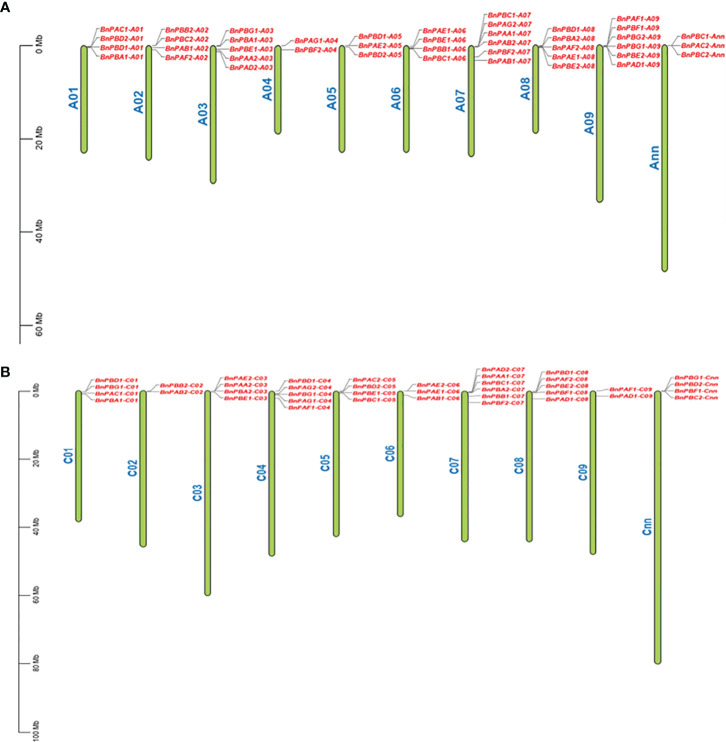
Distribution of 82 *BnPA* and *BnPB* genes on 20 chromosomes of *B. napus* belonging to the A **(A)** and C **(B)** subgenomes. On each chromosome, gene names are given on the right side. A scale of physical positions in megabases (Mb) is shown on the far left.

All 82 *BnPA* and *BnPB* genes were physically mapped to 18 individual chromosomes of *B. napus* along with two additional Cnn and Ann chromosomes. The maximum number of six genes were located on each A07, A09, and C07, while the minimum number of two genes each was located on A04, A02, and C09. All the genes were located in the terminal and subterminal positions (**Figure 2**). The tree involving *B. napus* genes along with genes from other taxa was developed using the Ensembl Plants Compara pipeline (**Supplementary Figure S1**). This was done to examine orthology and paralogy among 35 *BnPA* and 47 *BnPB* genes.

Detailed information on the orthologous and paralogous relationships of the *BnPA* and *BnPB* genes with different species is given in **Supplementary Table S1**. Orthologs and paralogs among Brassica, Arabidopsis and rice 20S proteasome genes have been investigated using the order of genes of 14 subunits (7-α and 7-β). Out of the 24 genes in Arabidopsis (13- α and 11- β), the number of duplicated genes was 10 and those that remained single were 4. Out of the 23 rice genes (13-α and 10-β), duplication was observed in seven genes, triplication in 1, and the rest 6 genes remained single (**Supplementary Table S1**). The observed pattern of duplicated genes in *B. napus* was similar to that in Arabidopsis and rice for the corresponding *PA/PB* genes. The orthologs were distinguished from paralogs using Ensembl Plant Compara.

#### 3.1.3 Synteny between *B. napus*, *A. thaliana*, and rice

**Figure 3:**
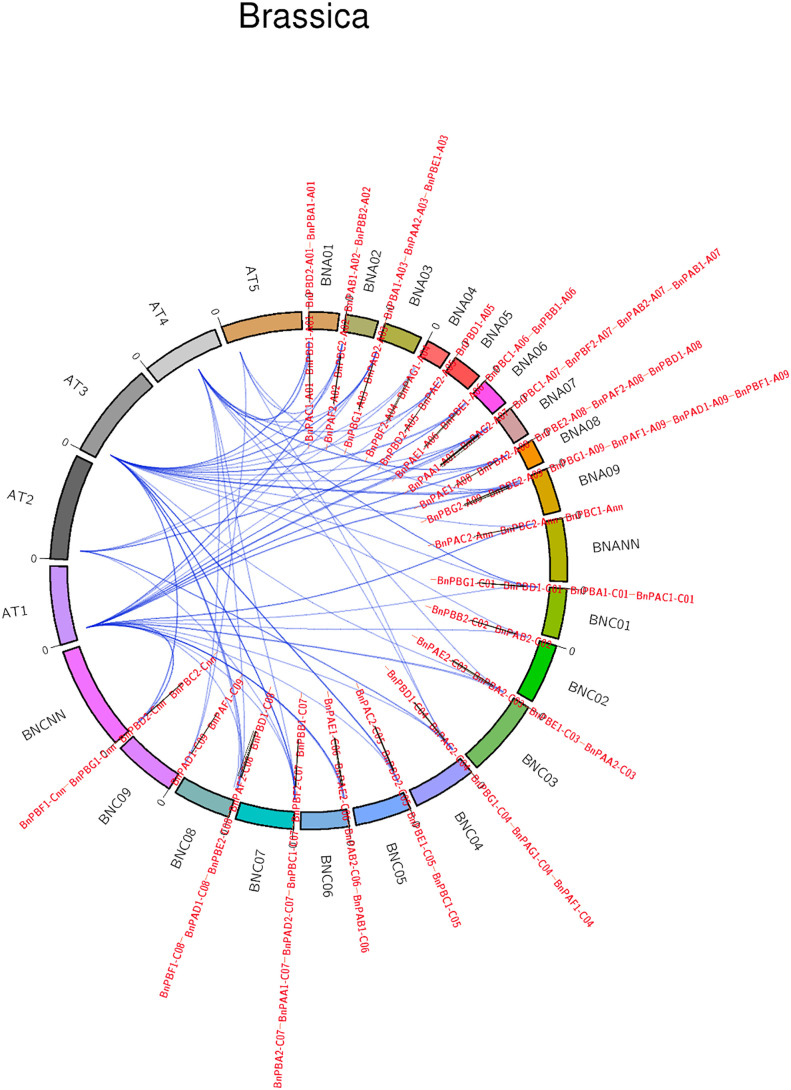
A circular map showing synteny among PA and PB genes of *B. napus*, *A. thaliana* and rice (name of chromosomes given outside the circle, name of genes given outside and inside the circle).

Synteny analysis of 82 *BnPA* and *BnPB* genes with the corresponding Arabidopsis genes was highly conserved (**Figure 3**). The collinearity of *BnPA* and *BnPB* genes with Rice was negligible; however, collinearity with *A. thaliana* was found with only one gene, i.e., *BnPBC2*-Cnn with *At1*.

#### 3.1.4 SSRs in *BnPA* and *BnPB* genes

A total of 66 SSRs were identified in 45 (54.87%) of the 82 genes. Of these, 28 SSRs were identified in 16 *BnPA* genes and 38 SSRs were identified in 29 *BnPB* genes. The SSR numbers vary per gene. There were only six SSRs in *BnPAE1-A08*, only four SSRs in *BnPBB1-C07*, three SSRs in two genes (*BnPAE2-C03*, *BnPBD2-C05*), and two SSRs each in nine genes (*BnPAA2-A03, BnPAE1-C06, BnPAE2-A05, BnPAF1-C09, BnPAG2-C04, BnPBA1-A01, BnPBC1-A06*, *BnPBD2-Cnn* and *BnPBF2-A07*), whereas single SSRs were present in the remaining 32 genes (**Supplementary Table S3**). SSRs with dinucleotide motifs (20) are more frequent followed by tetranucleotide (10) and hexanucleotide (10), trinucleotide and mononucleotide (9), pentanucleotide (5) and heptanucleotide (3) motifs.

#### 3.1.5 Analysis of promoter and cis-acting regulatory elements

Cis-acting regulatory elements present 1500 bp upstream of the 5’ promoter sequence were studied in each of 82 *BnPA/BnPB* genes. It was observed that the promoter region of all the genes contains many light-responsive elements along with phytochrome and other cis-acting elements. The maximum number of genes was for light, methyl jasmonate (MeJA), and endosperm elements (**Supplementary Figure S2**).

#### 3.1.6 MicroRNAs and their targets in *BnPA* and *BnPB* genes

A total of 44 microRNAs (miRNAs) were found. These mi-RNAs had their targets in 21 genes (8 *BnPA* and 13 *BnPB*). The maximum number of target sites i.e., six microRNAs were available for each of two genes (*BnPAC1-A01* and *BnPBD2-A01*). The targets for *BnPBD1-C08, BnPBD1-A08* and *BnPBD2-Cnn* were available for four miRNAs. The target for *BnPAF2-C08* was available for three miRNAs. Similarly, the targets for *BnPBA1-A01* and *BnPBA2-C07* were available for two miRNA each. The target for the remaining 13 miRNAs was available in different *BnPA* and *BnPB* genes distributed on several Brassica chromosomes (**Supplementary Table S4**).

### 3.2 *In silico* expression analysis of *BnPA* and *BnPB* genes

The expression pattern of genes was studied under normal and stress conditions. Under normal conditions, two different developmental stages (germination and seedling) for six different organs (seed, cotyledon, hypocotyl, radicle, leaf, and root) were examined. Under both abiotic and biotic stress conditions, two different developmental stages (seedling both flowering) were examined.

#### 3.2.1 Tissue-specific expression under normal conditions

**Figure 4:**
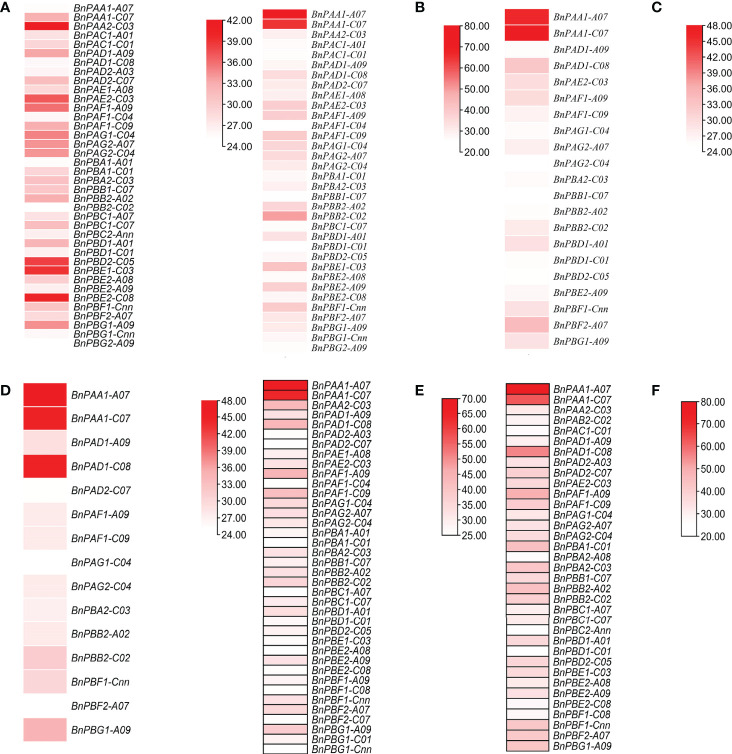
Expression profile of *BnPA* and *BnPB* genes at two developmental stages in six different tissues, **(A)** germination (cotyledon), **(B)** germination (seed), **(C)** germination (hypocotyl), **(D)** seedling (leaf), **(E)** germination (radicle) and **(F)** seedling (root), under normal conditions in *B. napus*. The expression data are represented in the form of FPKM values.

Under two different developmental stages (germination and seedling), the six different organs (seed, cotyledons, hypocotyl, radical, leaf, and root) showed variation in expression (**Figure 4**). Out of 82, 18 genes showed higher expression in the two stages and the expression data are provided in the form of FPKM values.

#### 3.2.2 Abiotic stresses

**Figure 5:**
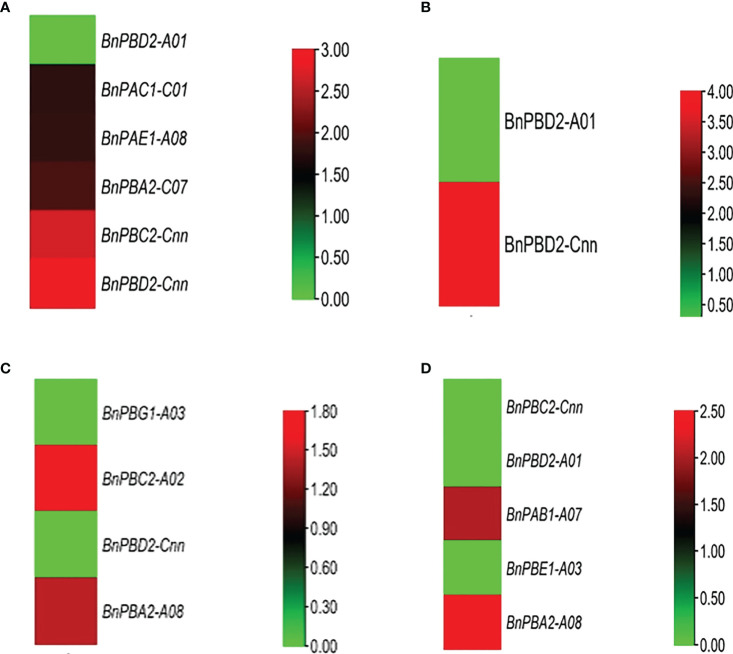
*In silico* expression profiling of *BnPA* and *BnPB* genes at seedling and flowering stages under abiotic stresses **(A)** cold and **(B)** drought and biotic stresses **(C)** *Leptosphaeria maculans* and **(D)** *Sclerotinia sclerotiorum*. The expression data of control and stress conditions are shown in the form of relative fold change (FC).

Cold stress. We observed two genes under 24 h cold stress (seedling and flowering stages) whose expression was upregulated i.e., >1.70-fold change (FC). During this same duration, the expression of 33 genes ranged from 0.00 to 0.99 FC i.e., the expression of these genes in response to cold stress was very poor (**Figure 5A**).

Drought stress. Only one gene showed high expression (FC>1.70) under 24 h drought stress (seedling and flowering stages), whereas 62 genes showed their expression from 0.00 to 0.99 FC (**Figure 5B**).

Heat Stress. Under 24 h of heat stress, all genes (seedling and flowering stages) showed expression FC<1.70. However, the expression of 57 genes ranged from 0.00 to 0.80.

As a result, we concluded that in *B. napus*, there are only five genes that can tolerate cold stress and there is only one gene that is expressed under drought conditions. Moderate to poor expression of genes was observed under heat stress conditions.

#### 3.2.3 Biotic stresses

Under biotic stress in *B. napus*, 20S proteasome genes were expressed against two fungal pathogens *Leptosphaeria maculans* (blackleg) and *Sclerotinia sclerotiorum* (white mold), at the seedling and flowering stages, respectively. After 264 h of *L. maculans* infection at the seedling stage, one gene with FC>0.99 was expressed against fungal infection (**Figure 5C**). Relative to the control, 1 gene was stimulated (FC>1.70) after 72 h of infection at the seedling stage. At the flowering stage, BnPAB1-A07 and BnPBA2-A08 genes were expressed with FC>0.99 after 48 h and 96 h of *S. sclerotiorum* inoculation, respectively (**Figure 5D**).

### 3.3 Analysis of Proteins

#### 3.3.1 Characterization of BnPA and BnPB proteins

The molecular weight of BnPA and BnPB proteins ranged from 9510.19 to 82935.95. the isoelectric point (PI) ranged from 4.65 to 9.93. The total acidic and alkaline proteins were 71 and 11 respectively. The proteins (41) with a lower aliphatic index i.e., 67.30 to 98.52 were unstable, whereas the remaining proteins (41) had a higher aliphatic index of 79.60 to 100.36 and were stable. The grand average of hydropathy (GRAVY) ranged from -0.669 to 0.093 (**Supplementary Table S5**).

#### 3.3.2 Functional domains and motifs of BnPA and BnPB proteins

The number of amino acids in 82 BnPA/BnPB proteins varied from 82 amino acids (aa) (BnPBC2-Cnn) to 732 aa (BnPAE1-A06), the mean of which was 251.85 aa (**Supplementary Table 2**). The length of α-subunits ranged from 201 to 732 aa, and the β-subunits ranged from 82 to 525 aa (**Supplementary Table 2**). A total of 10 separate motifs are given in **Figure 1**. The logo of 10 separate motifs and the associated amino acids identified in the BnPA and BnPB proteins are provided in **Supplementary Figure S3**. The list of identified motifs along with their sequence and e-value are given in **Supplementary Table S7**.

#### 3.3.3 Sequence alignment and assessment of conserved amino acids

The percent similarity among the α-subunit of *B. napus* was 30 (**Supplementary Table S8**) whereas in the β-subunit it was 20.72 (**Supplementary Table S9**). The similarity percentage between the α and β- subunits of *B. napus* was 21.33. The percentsimilarity among *B. napus* and Arabidopsis *BnPA* proteins was 91.06 (**Supplementary Table S8**) and for BnPB protein was 99.14 (**Supplementary Table S9**). High amino acid similarity was observed among the α and β-subunits of *B. napus*, Rice, and Arabidopsis (**Supplementary Figures 4a, b**). A high conservation pattern of 31 amino acids (aa) of α-subunits and 15 β-subunits was found among *B. napus*, rice, and Arabidopsis. These 31 (α-subunits) and 15 (β-subunits) residues also had the highest MI (**Supplementary Figures S5, S6**).

#### 3.3.4 Localization of subcellular proteins and their functions

**Figure 6:**
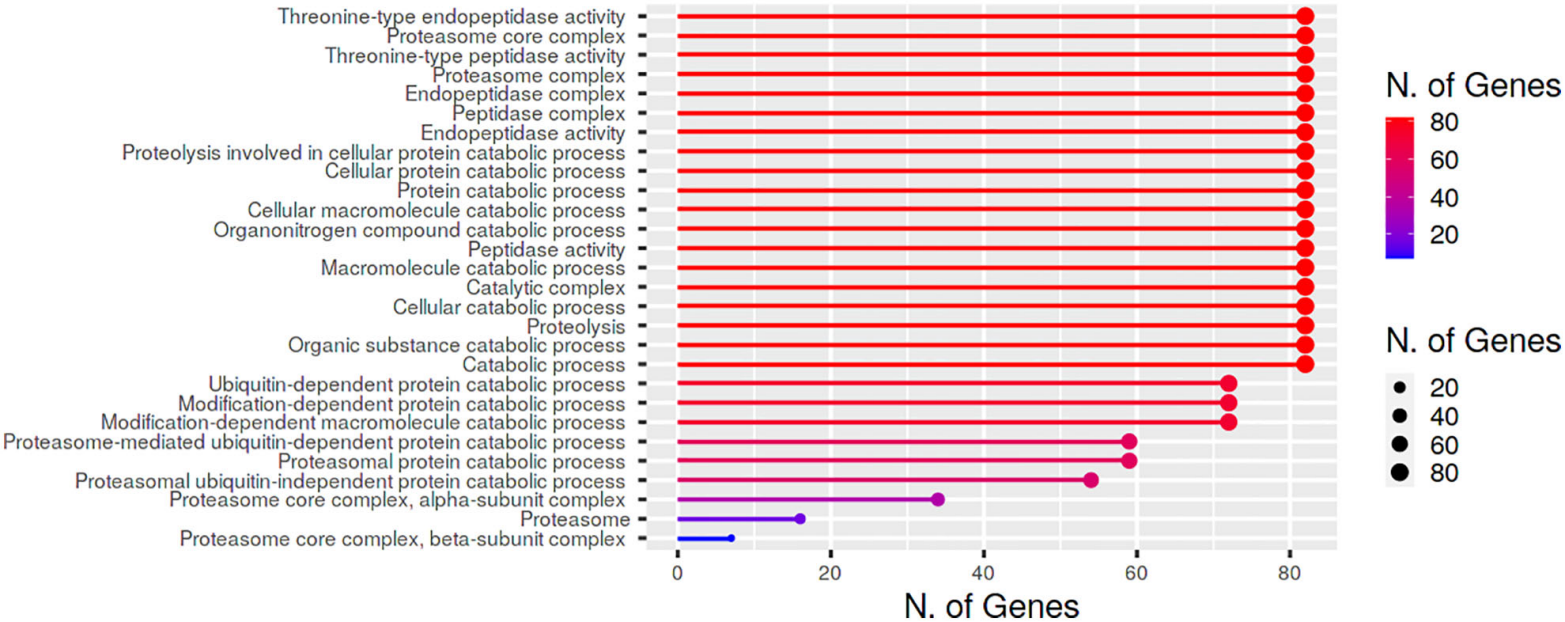
Cellular location and biological/molecular functions of BnPA/BnPB proteins in *Brassica napus*.

Gene ontology analysis and functional annotation suggested that *BnPA and BnPB* proteins have proteolytic functions and other molecular functions such as threonine type endopeptidase activity, threonine type peptidase activity, peptidase activity and endopeptidase activity (**Figure 6**).

#### 3.3.5 Protein structures

The secondary structure of all the proteins was compared. It has been found that the secondary structure is dominated by α-helices followed by random coils (**Supplementary Table S10**). The latter form irregular structures that permit polypeptide chains to fold uniquely. Proteins such as *BnPA and BnPB* tend to form a highly stable structure. *In-silico* 3D structures were determined for 20 (24.39%) BnPA and BnPB proteins with similarity ranges of 10.85-100% with the corresponding template of Arabidopsis (**Supplementary Figure S7**). The GMQE for these 20 proteins ranged from 0.59 to 0.82. A high-quality protein model was suggested by it. Q-mean value ranged from 0.73 ± 0.06 to 0.82 ± 0.06. The similarity between the protein model and reference (Arabidopsis) proteins ranged from 42 to 69%. The quality factor was determined with the help of ERRAT and ranged from 88.2096 to 100%. The 3D-1D score was determined with the help of VERIFY 3D and ranged from 77.95 to 99.58% (**Supplementary Table S11**)

#### 3.3.6 Alignment and functional annotation of 3D-structure

**Figure 7:**
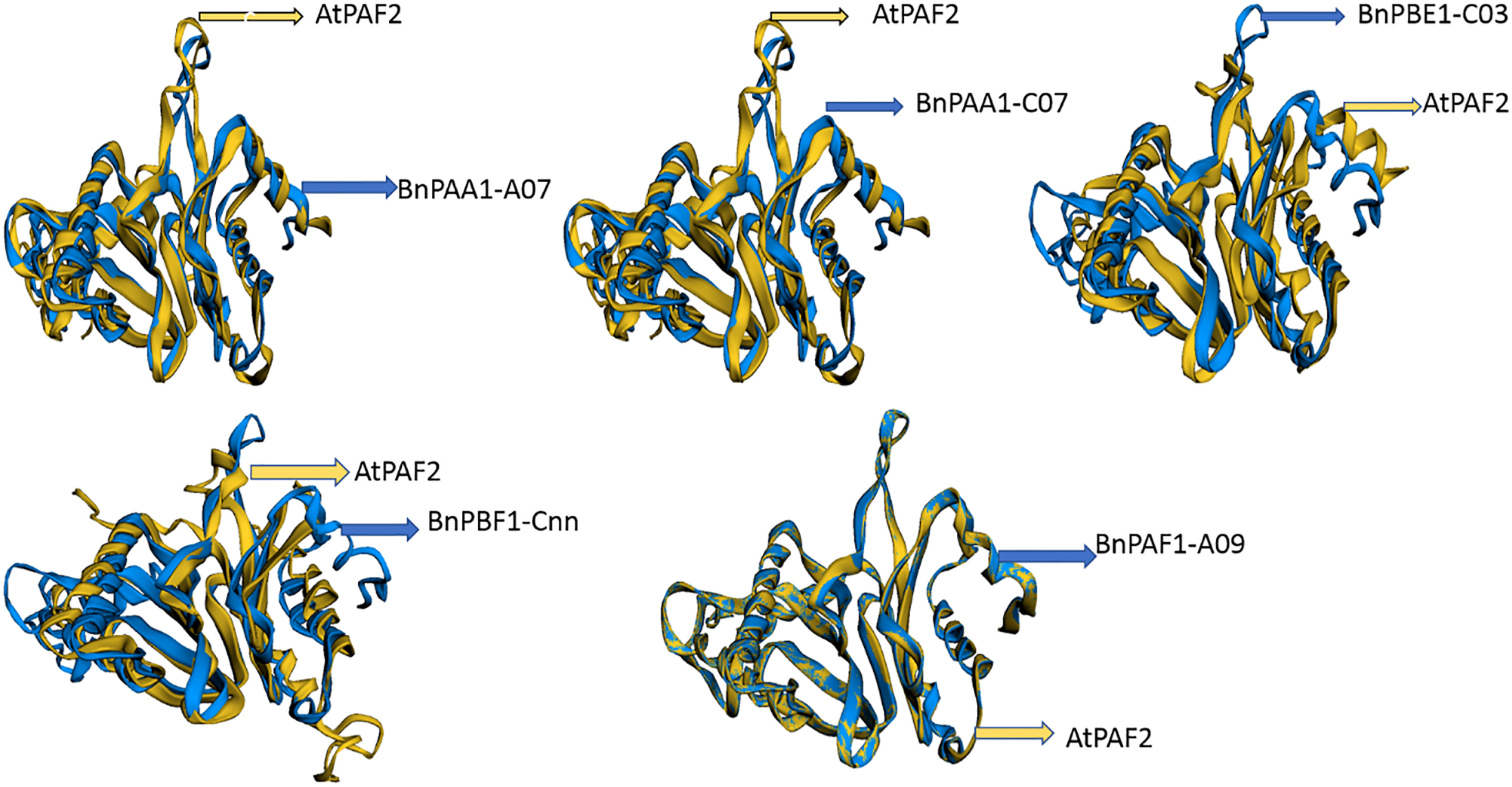
Representative figure showing the superimposed structure of the predicted 3D structure of BnPAA1-A07, BnPAA1-C07, BnPAF1-A09, BnPBE1-C03 and BnPBF1-Cnn proteins over the 3D structure of *Arabidopsis* PAA (AtPAF2) proteins.

The 3D protein structures (with minimum energy) of *B. napus* were superimposed with the reference protein (3D) of Arabidopsis (**Figure 7** and **Supplementary Table S12**). The 3D structure of five (BnPAA1-A07, BnPAA1-C07, BnPAF1-A09, BnPBE1-C03, and BnPBF1-Cnn) proteins showed a similarity of 33 to 98% with the corresponding 3D structure of the AtPAF2 protein with RMSD values in the range of 0.04 to 2.66 Å.

### 3.4 Phylogenetic analysis

**Figure 8:**
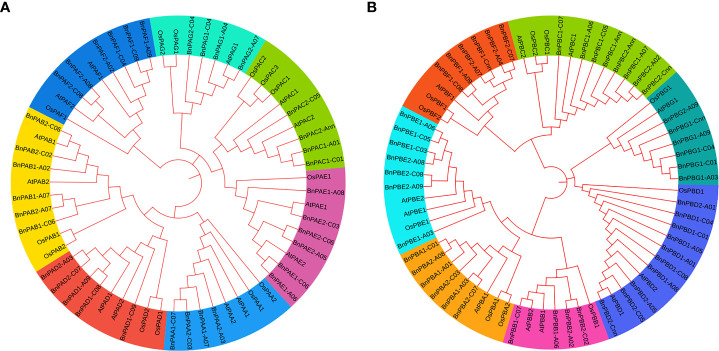
Phylogenetic tree constructed using protein sequences of **(A)** α-subunits and **(B)** β-subunits belonging to three plant species (*A. thaliana*, *O. sativa* and *B. napus*). Seven different colors in the tree represent seven different clades.

The amino acid sequences of *B. napus*, *A. thaliana* and *O*. *sativa* for α- (PA) and β-subunits (PB) were utilized separately to construct a phylogenetic tree. Seven clades that belong to 1 to 7 subunits were observed in each phylogenetic tree. These clades showed similarities with the conserved motifs (**Figure 1**). It was interesting to note that the orthologs of all three taxa belong to each of the α and β-subunits and formed seven clades in both phylogenetic trees. In the α-subunit tree, 7-10 orthologs formed clades (**Figure 8A**) whereas, in the β-subunit tree, 7-13 orthologs formed clades (**Figure 8B**).

## 4 Discussion

Plant genome sequencing has been utilized to study genes related to various developmental stages and stress tolerance in many crops (Lu et al., 2019; Mehla et al., 2022). Crops whose genomes have not been sequenced are receiving benefits from those crops whose genomes have been sequenced. *A. thaliana* and *O. sativa* (Rensink and Buell, 2004) have been utilized for such studies.

### 4.1 Identification of *BnPA* and *BnPB* genes

The present study is the first report in *B. napus* on phylogenomic analysis for identifying and describing genes that encode for various subunits (α1-7 and β1-7) of the 20S proteasome. In this study, 82 *BnPA* and *BnPB* genes of *B. napus* were arranged into seven different α and β types of 20S proteasome. The sub-genomes A and C contain 42 and 40 genes, respectively. When compared to previously identified *A. thaliana* (*AtPAA-AtPAG and AtPBA-AtPBG*) and rice genes (*OsPAA-OsPAG and OsPBA-OsPBG*) (Fu et al., 1998; Sharma et al., 2022), a fairly large number of these genes were detected in *B. napus*. These findings may justify the fact that *B. napus* has a large genome and evolved with a higher ploidy level. The presence of two more genes in genome A of *B. napus* may be due to duplication, and a lower number of genes in genome C than genome A may be due to the loss of the gene. In the majority of the *BnPA* and *BnPB* genes, the structural pattern of exons and introns was found to be similar; however, in some cases, this similar pattern deviates. This deviation in gene structure may be due to the loss/gain of introns during the course of evolution (Rogozin et al., 2003; Yu et al., 2019). In cDNA sequences, this may be due to differences in intron number and size located in *BnPA* and *BnPB* genes. In addition, the variation in the length of UTRs present on the borders of cDNA may be due to differences in the length of cDNA sequences. The phylogenomic analysis of 82 genes revealed seven clades of each α (BnPAA-BnPAG) and β (BnPBA-BnPBG) subunit. The different α and β subunits of *B. napus* varied from each other but they showed similarities with Arabidopsis, a close relative of *B. napus*. This means that the 20S proteasome might have not changed after the divergence of these two taxa due to common ancestry. The distribution pattern of *BnPA* and *BnPB* genes on A-09, C-09, Ann and Cnn differed. In our findings, we found an uneven distribution of genes in genomes A and C. The general pattern is four genes per chromosome but in some cases, there is a deviation from this pattern. This deviation may be due to gene duplication or gene loss (Clavijo et al., 2017; Yu et al., 2019). The difference in the distribution of orthologous genes in the A and C genomes may be due to inversion and translocation.

### 4.2 Number of genes in Arabidopsis, rice and *B. napus*

**Figure 9:**
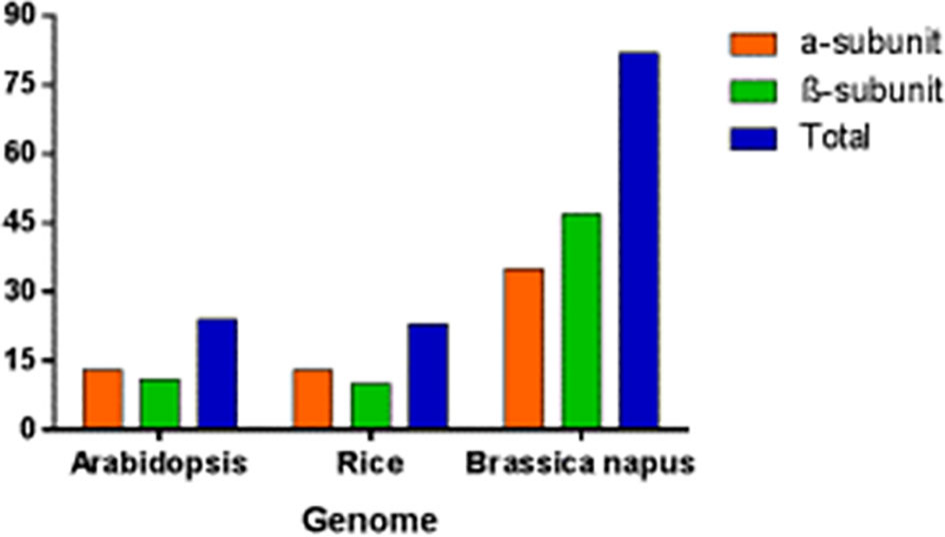
Genes encoding seven α and seven β subunit proteins of the 20S proteasome in Arabidopsis, rice and *B. napus*.

In *B. napus*, 82 *BnPA* and *BnPB* genes of 20S PA and PB have been reported so far which represent the highest numbers in the plant kingdom. This number is thrice the number reported in Arabidopsis and rice (**Figure 9**). The number of genes in A and C suggests that different genes have undergone duplication (paralogy) and speciation events (orthology). In Arabidopsis, out of 24 genes (**Supplementary Table S2**), instead of 14 genes as published in earlier studies (Fu et al., 1998), 13 genes with duplications for all α-subunits except *AtPAG* (α-7), whereas 11 genes with duplications in only 4 β-subunits, *AtPBB* (β-2), *AtPBC* (β-3), *AtPBD*(β-4), and *AtPBE* (β-5), have been reported (Parmentier et al., 1997; Fu et al., 1998).

Similarly, a phylogenomic survey of rice determined 23 genes instead of 14, as published in previous studies (Sassa et al., 2000). The duplications and heterogeneity have also been resolved in other organisms by phylogenomic surveys. A phylogenomic survey in *B. napus* revealed that the α-subunit included 35 genes with duplications except for *BnPAC1, BnPAA2, BnPAD2, BnPAG1, BnPAG2, BnPAA1*, and *BnPAC2*, and the β-subunit include 47 genes with duplication except for *BnPBB2, BnPBB1*, and *BnPBG2* (**Supplementary Table S2**). The α-genes found with maximum duplication are *BnPAB1, BnPAF2, BnPAE2, BnPAE1, BnPAB2, BnPAF1*, and *BnPAD1*. Similarly, the β-genes found with maximum duplication are *BnPBD1, BnPBG1*, and *BnPBC1*.

### 4.3 Duplication and analysis of synteny in *BnPA* and *BnPB* genes

The present results on the 20S proteasome gene family of *B. napus* showed that in most cases duplication events have occurred in both the A and C genomes. The duplication events are from 1 (*BnPBG2-A09*) to 6 (*BnPBD1*). *BnPBD1* was reported on A01, A05, A08, C01, C04, and C08 suggesting maximum duplication (paralogy). These duplications may be due to translocation during the course of evolution. The absence of any duplication in *BnPBG2-A09* may be due to deletion. We have checked the synteny of all five chromosomes of Arabidopsis with all proteasome genes of *B. napus*. It was found that 81 genes of *B. napus* were syntenic with the genes on the chromosomes of *A. thaliana*. One gene of *B. napus* (*BnPAD1-C09*) was found to be nonsyntenic with *A. thaliana*. This could be due to deletion. Chromosome 1 and chromosome 3 of *A. thaliana* showed synteny with 29 genes of *B. napus*, whereas the least synteny was observed with chromosome 5, i.e., only with 4 genes of *B. napus*. All the genes on the chromosome of *O. sativa* were nonsyntenic.

### 4.4 Analysis of promoter sequences in *BnPA* and *BnPB* genes

The cis regulatory elements, present mostly near the start codon, are noncoding in nature and under different environmental conditions, they modulate gene expression in response to different transcription factors. Gene regulation is determined by cis regulatory elements at the basic level (Mondal and Das, 2020). The identified cis regulatory elements that spanned the promoter of 82 proteasomal genes were found to be associated with development and stress responses (light responsive, endosperm, and MeJA signaling) in Brassica (**Supplementary Figure S2**). Thus, it is evident that MeJA regulates plant growth and environmental stress (Wang et al., 2020) and defense against pathogens and herbivores (Fürstenberg-Hägg et al., 2013). There is a large number of evidence suggesting that hormonal signals affect the expression of the 20S proteasome gene. This expression leads to biotic and abiotic stresses (Kurepa et al., 2009). The presence of light-responsive elements indicates that they are required for light-dependent transcriptional regulation (Hiratsuka and Chua, 1997; Mondal et al., 2022). Similarly, the presence of endosperm elements is expressed during developmental stages. A large number of GBREs along with other cis-elements respond to phytohormones such as MeJA, and ABA available in *BnPA* and *BnPB* genes may control the expression of genes (*BnPA* and *BnPB*) under heat stress (Cao et al., 2015).

### 4.5 SSR with SSRs and miRNA with miRNAs

In this study, out of 82 genes, we found 66 SSRs in only 45 genes. This suggests that a fraction of genes in a gene family contain SSRs. The structural and functional aspects of SSR have been reported in a large number of genes (Li et al., 2004; Gupta and Rustgi, 2004; Varshney et al., 2005). The frequency of dinucleotide was highest (20). It was followed by tetranucleotide and hexanucleotide. Such occurrence is unusual because trinucleotide repeats are generally found most frequently in comparison to other SSRs (Gupta and Varshney, 2000). The SSR reported in the gene that encodes the α and β-subunits of proteasome core particles, shows polymorphism which can be helpful to develop functional molecular markers. Such a marker can be used to improve tolerance against different physiological stresses in the plant. The absence of transposable and retroelements in the genes studied indicates the nonexpression of the 20S proteasome family in *B. napus*.

The mi-RNAs are small noncoding RNAs that have a regulatory function inside the cells at the post-transcriptional and translocation levels. These cause the degradation of targets of the gene (Budak & Zhang, 2017; Liu et al., 2017). In this study, 44 miRNAs involving sequences of *BnPA* and *BnPB* genes were identified. The target sites for these miRNAswere found only in 21 genes (8 *BnPA* and 13 *BnPB*). Among the predicted miRNAs, candidates of bna-miR166 (miR166 a-f; each 21 nucleotides long) were detected in *BnPAC1-A01* and those of miR160 (miR160 a-d) were detected in *BnPBD-C08, BnPBD1-A08, BnPBD2-A01*, and *BnPBD2-Cnn* (**Supplementary Table S4**). Micro-RNA166 (miR166), a highly conserved family of miRNAsis involved in several cellular and physiological processes in plants (Li et al., 2017), such as drought stress (Kantar et al., 2011), cold stress (Liu et al., 2008) and heat stress (Khraiwesh et al., 2012). F-box/SCF is encoded by miR2111, which is involved in ubiquitin-mediated proteolysis and then in the adaptive response of Pi-deficiency (Kumar et al., 2017). miR160 has a role in both abiotic and biotic stress responses in Arabidopsis and maize (Zhang et al., 2009; Moldovan et al., 2010). miR160a is involved in regulating auxin response genes, club root development, and disease modulation (Verma et al., 2014). In this way, the function of miR166 and miR160 is understood in plants. The presence of miR166 and miR160 in *B. napus* may be significant for future studies on the biological role of these miRNAs.

### 4.6 Structural and functional features of *BnPA and BnPB* proteins

The predicted proteins of *BnPA* and *BnPB* genes were found to be variable and, differed in theprotein length of similar genes from *A. thaliana* and *O. sativa*. The number of amino acids in BnPA and BnPB varies from 82 aa (*BnPBC2-Cnn*) to 732 aa (*BnPAE1-A06*). This length is more variable than those of rice proteins (237 to 256 aa). Similarly, the length of proteins (199 to 298 aa) of *A. thaliana* is the least variable (Town et al., 2006). The GRAVY ranged from -0.669 to 0.093. This suggests the hydrophobic nature of these proteins. Due to this property, there will be proper folding of proteins to keep them stable and biologically active.

The *BnPA* and *BnPB* genes encode proteins that contain 10 different motifs. Similarly, 10 motifs are present in the database for Arabidopsis and rice (Parmentier et al., 1997; Fu et al., 1998; Sassa et al., 2000). The length of the individual motif varied from 15 aa (motif 3) to 41 aa (motif 10). Motifs 1, 4, 5, and 6 are concerned with catabolic processes at the cellular level (https://www.ebi.ac.uk/interpro/result/InterProScan/iprscan5-R20211209-091508-0331-1308065-p2m/). The remaining 6 motifs were found to be novel and need to be characterized at the molecular level. Earlier studies reported that in eukaryotes, proteins of the 20S proteasome could be in the nucleus and cytoplasm, and the same case may be true for BnPA and BnPB proteins (Fu et al., 1998). A single specific domain of α-type (α 1-7) or β-type (β 1-7) is present in all 82 BnPA and BnPB proteins. Of these, 35 BnPA proteins contain a single α-type domain and 47 BnPB proteins contain a single β-type domain (**Supplementary Table 2**). Similar findings have been observed in yeast, Arabidopsis and rice (Parmentier et al., 1997; Groll et al., 1997; Fu et al., 1998; Arias and Pires, 2012). A majority of aa residues (>90%) that fall in the most favored region were shown by modeled 3D structures of 20 BnPA/BnPB proteins. This suggested that the predicted models are reliable (Laskowski et al., 1993; Kumar et al., 2018; Batra et al., 2019). The predicted 3D structures of BnPA and BnPB proteins may be helpful for the initial understanding of molecular functions.

### 4.7 Phylogenetic analysis of BnPA and BnPB protein sequences

The different orthologs of the α and β-subunits of 20S proteasome were examined, and phylogenetic trees were constructed. The orthologs of Brassica, Rice and Arabidopsis that belong to different α and β-subunits were depicted into seven clades for the α and β subfamilies. Similar findings have been reported for yeast, Arabidopsis and rice proteins (Fu et al., 1998; Sassa et al., 2000). These findings suggest that the α and β-subunits of *B. napus* have a higher level of similarity with those of Arabidopsis and rice.

### 4.8 Digital expression analysis of *BnPA* and *BnPB* genes

All 20S proteasome genes and core particles are involved in plant responses to different biotic and abiotic stresses (Xu and Xue, 2019). This is due to different signaling molecules that control the development of plants under different stress conditions (Livneh et al., 2016). Earlier a few studies (Fu et al., 1998; Li et al., 2015) examined digital expression analysis of the 20S proteasome gene in a plant system. The present study is the first report in *B. napus* on phylogenomic analysis and digital expression of the 20S proteasome gene under normal tissue-specific and stress conditions in the germination and seedling stages. This was done using a transcriptome database in which approximately 18/82 genes showed higher expression at different germination and seedling stages of *B. napus* in root, seed, leaf, cotyledon, hypocotyls, and radicle (**Figure 4**). A greater mRNA level expression for six 20S proteasome subunit genes compared to β-tubulin in flower and fruit tissues of Arabidopsis has been observed *via* RNA blot analysis (Fu et al., 1998). Under 24 h of abiotic stress (cold, drought, and heat), two genes for cold and one gene for drought exhibited high expression at the seedling and flowering stage. No gene was reported for heat. However, in wild rice, the *OgTT1* gene was reported under heat stress conditions (Li et al., 2015). Under 264 h and 72 h of biotic stress (*L. maculans)* as many as one gene exhibited higher expression at the seedling stage. Under 96 h, 48 h and 24 h of biotic stress (*S. sclerotiorum)* as many as 1, 1 and 0 genes respectively, showed high expression in the flowering stage. In this way, the 20S proteasome gene plays a significant role in the development of different plant organs such as roots, seeds, leaves, cotyledons, hypocotyls, and radicles, as well as in abiotic stresses (Xu and Xue, 2019). The functions of the aforementioned genes that showed differential expression under biotic and abiotic stresses may be investigated in future studies.

## 5 Conclusion

The present study is the first report in *B. napus* on phylogenomic analysis for identifying and describing genes that encode 35 *BnPA* and 47 *BnPB* genes of the 20S proteasome. The orthology and paralogy of *BnPA* and *BnPB* genes were inferred and identified on the basis of the pattern of speciation and duplication of genes in Arabidopsis and rice. A full-length 3D model was predicted for the proteins encoded by *BnPA* and *BnPB* genes. A number of *BnPA* and *BnPB* genes were found to be expressed in many organs of *B. napus* under normal as well as abiotic and biotic stress conditions. In this way, the present study provides much information that can be utilized for the development of climate resilient cultivars of *B. napus.*

## Author contributions

VM, SK and UK planned and guided the design of this study. VK, HS, LS, AT, PJ, PB, SJ and ID retrieved sequence data, conducted computational analysis. VK, HS, LS, AT, PJ and PB performed data visualization, and wrote the first draft of the manuscript. VM, RM, KS, SK, YS, SJ, ID and UK critically revised and edited the manuscript. All authors have read and agree to the final version of the manuscript.

## Acknowledgments

The authors thank the Head, Department of Botany, Chaudhary Charan Singh University, Meerut, India for providing the necessary facilities to carry out this study and Head of Department of Molecular Biology & Biotechnology, College of Biotechnology, Chaudhary Charan Singh Haryana Agricultural University, Hisar, India for providing the necessary facilities to carry out this study.

## Conflict of interest

The authors declare that the research was conducted in the absence of any commercial or financial relationships that could be construed as a potential conflict of interest.

## Publisher’s note

All claims expressed in this article are solely those of the authors and do not necessarily represent those of their affiliated organizations, or those of the publisher, the editors and the reviewers. Any product that may be evaluated in this article, or claim that may be made by its manufacturer, is not guaranteed or endorsed by the publisher.
