# Supplementary material for: Phylogenomic analysis of 20S proteasome gene family reveals stress-responsive patterns in rapeseed (Brassica napus L.)
Source: Front Plant Sci. 2022 Oct 31;13:1037206. doi: 10.3389/fpls.2022.1037206 (PMC9659873; doi:10.3389/fpls.2022.1037206)
Supplement: Supplementary file 2 [file DataSheet_2.docx]

**Phylogenomic Analysis of 20S Proteasome Gene Family Reveals Stress-responsive Patterns in Rapeseed (*Brassica napus* L.)**

Vivek Kumar^1^, Hemant Sharma^2^, Lalita Saini^1^, Archasvi Tyagi^1^, Pooja Jain^1^, Yogita Singh^3^, Priyanka Balyan^4^, Sachin Kumar^2^, Sofora Jan^5^, Reyazul Rouf Mir^5^, Ivica Djalovic^6^, Krishna Pal Singh^7&8^, Upendra Kumar^3*^, Vijai Malik^1*^

^1^Department of Botany, Chaudhary Charan Singh University, Meerut – 250004 (UP) India

^2^Department of Genetics and Plant Breeding, Chaudhary Charan Singh University, Meerut – 250004 (UP) India

^3^Department of Molecular Biology & Biotechnology, College of Biotechnology, CCS Haryana Agricultural University, Hisar, India

^4^Department of Botany, Deva Nagri P.G. College, CCS University, Meerut-250001, India

^5^Division of Genetics and Plant Breeding, Faculty of Agriculture, SKUAST-Kashmir, Wadura-193201, India

^6^Institute of Field and Vegetable Crops, National Institute of the Republic of Serbia, Maxim Gorki 30, Novi Sad, Serbia

^7^Biophysics Unit, College of Basic Sciences & Humanities, GB Pant University of Agriculture & Technology, Pantnagar, 263145, India

^8^Vice-Chancellor’s Secretariat, Mahatma Jyotiba Phule Rohilkhand University, Bareilly-243001, India

*Corresponding authors

Dr. Upendra Kumar, Email: baliyan.upendra@gmail.com

Dr. Vijai Malik, Email: [gathwalajai@gmail.com](mailto:gathwalajai@gmail.com)


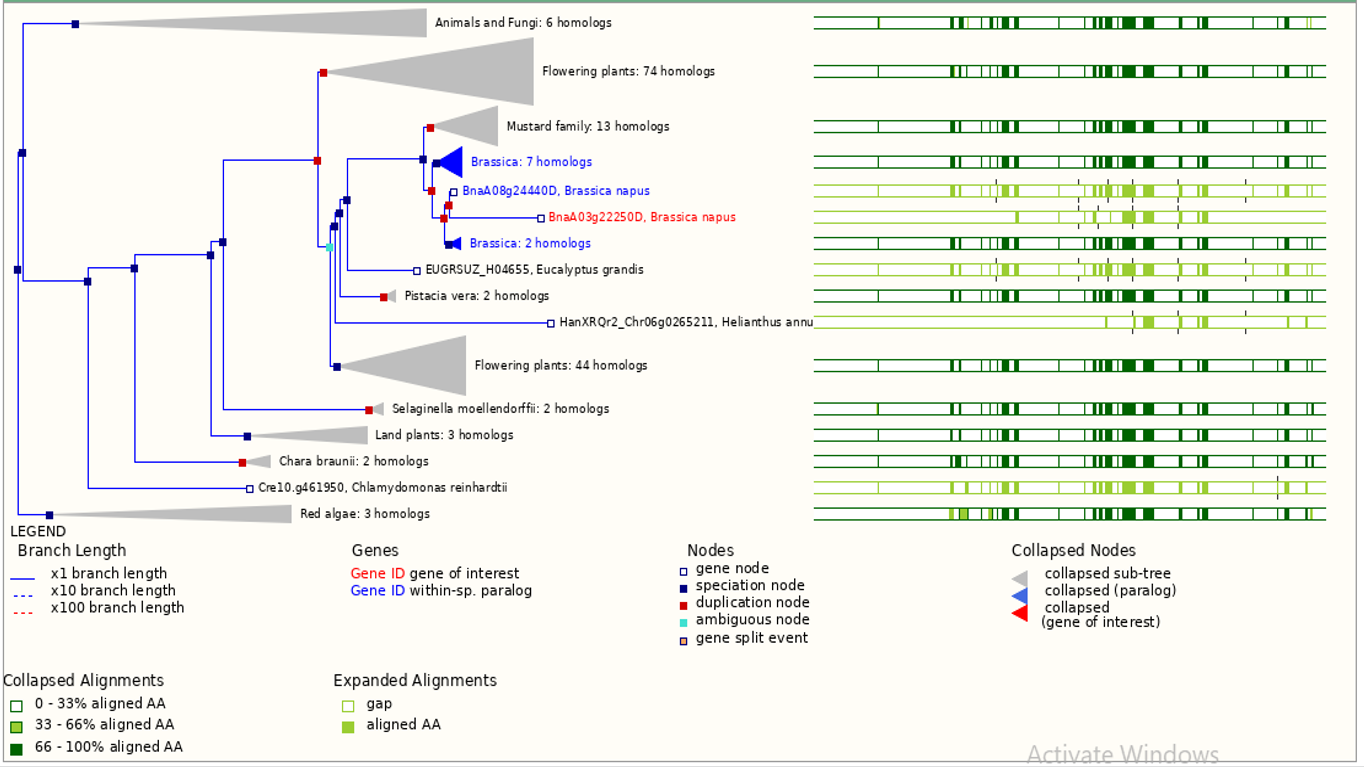


**Supplementary Figure 1.** Ensembl plants gene tree pipeline showing orthologous and paralogous relationship of *BnPA* and *BnPB* genes with other taxa. Duplication nodes are shown as red squares whereas speciation nodes are in blue.


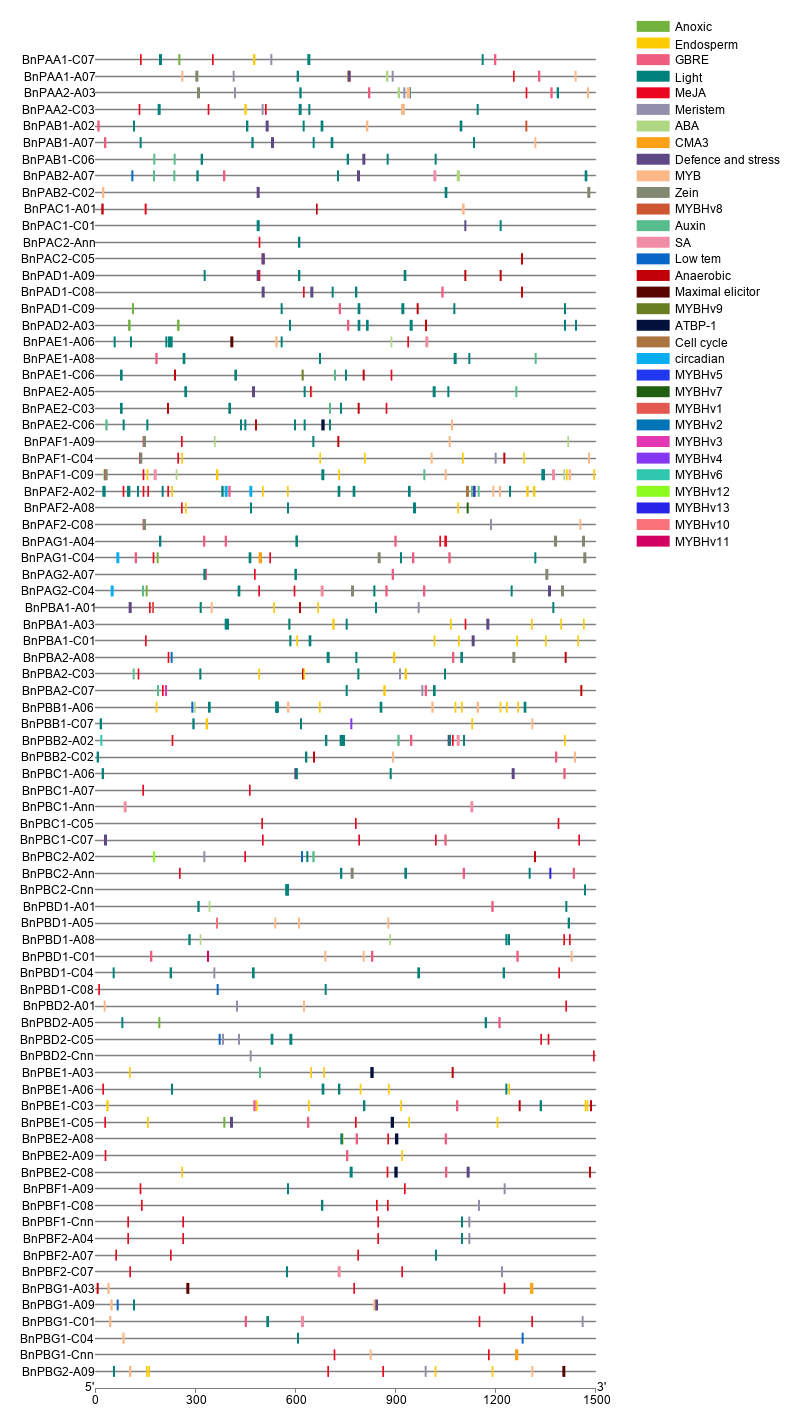


**Supplementary Figure 2.** The figure shows cis-regulatory elements with location in 82 *BnPA* and *BnPB* genes.

####
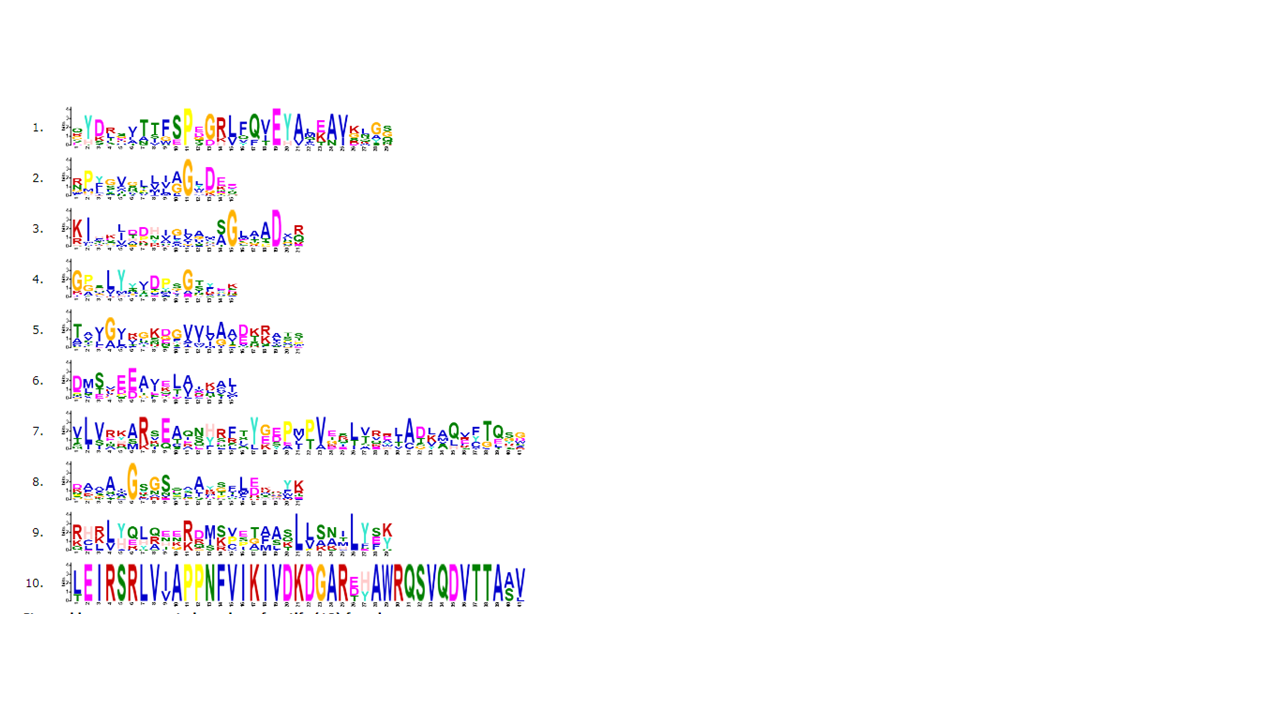


#### Supplementary Figure 3. Logo of each of the 10 motifs and associated amino acids identified in sequences of BnPA*/*BnPB proteins using MEME program (relative heights of letters indicate their frequencies and level of conservation). The X-axis represents the length of motif and Y-axis represents the sequence conservation per site (bit score) of each letter.


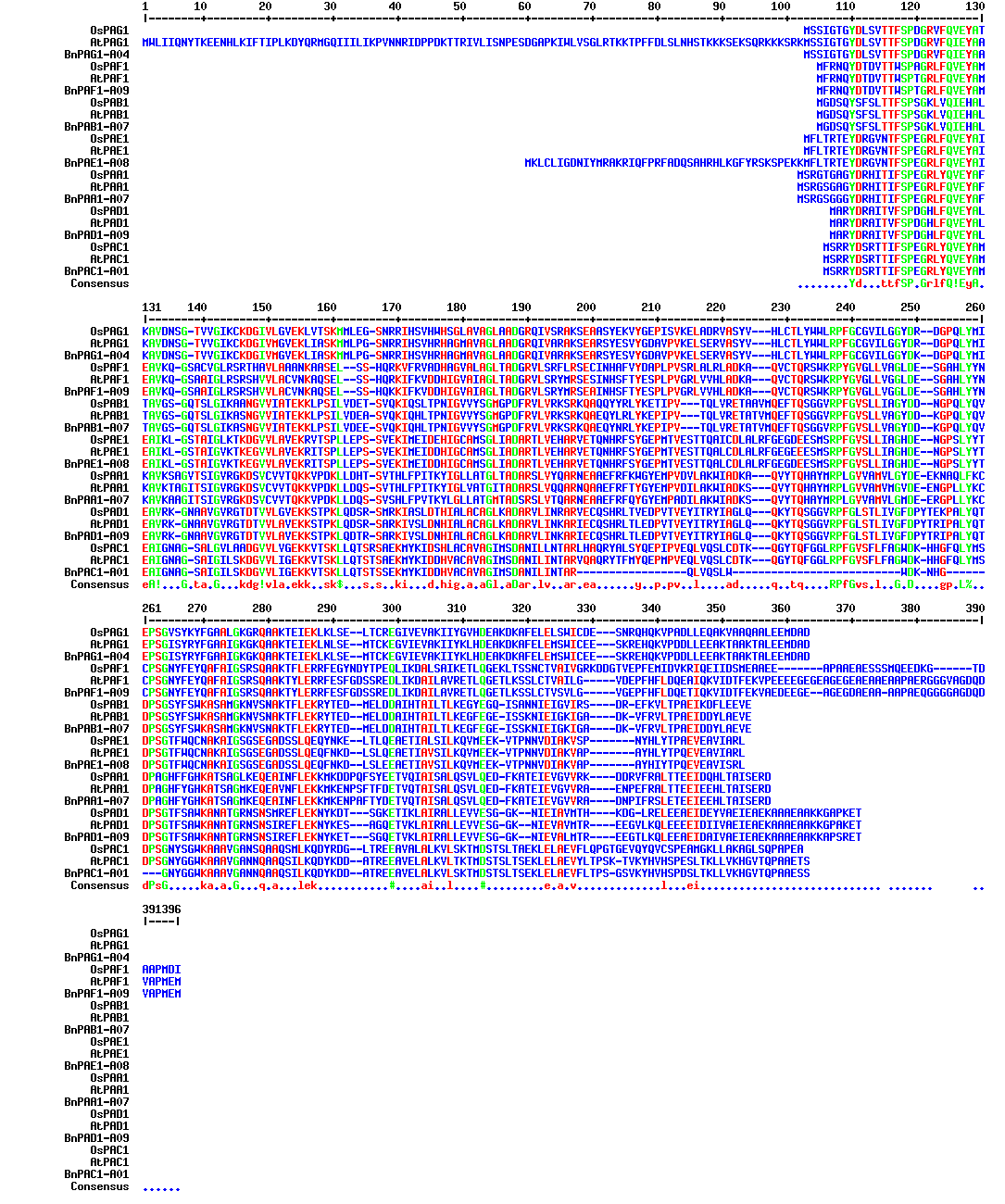
(a)


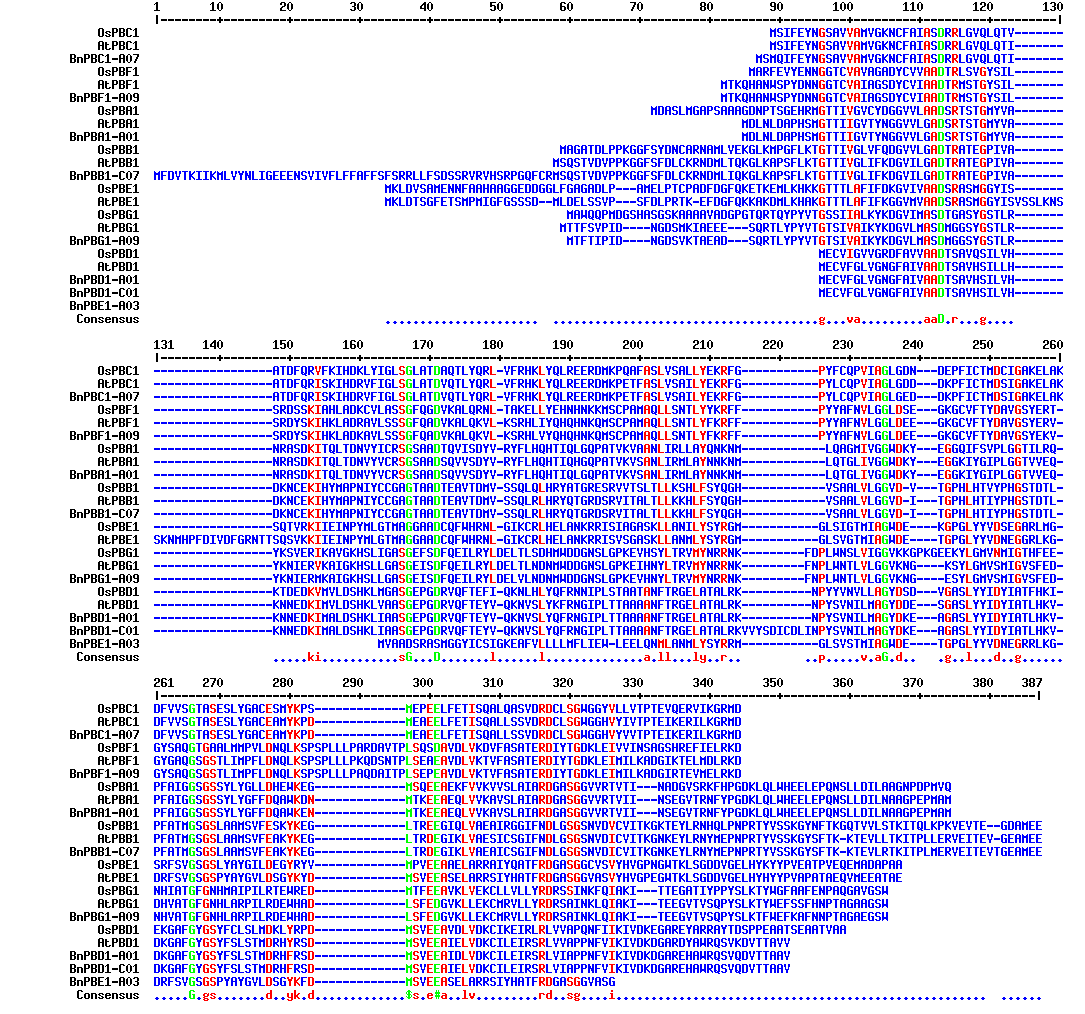


(b)

**Supplementary Figure 4.** Amino acid sequence alignments of the seven α (a) and seven β (b) subunits each of the of 20S proteasome of *Brassica napus*, rice and *Arabidopsis*. Conserved domains each α and β across three species are shown by different colours.

**
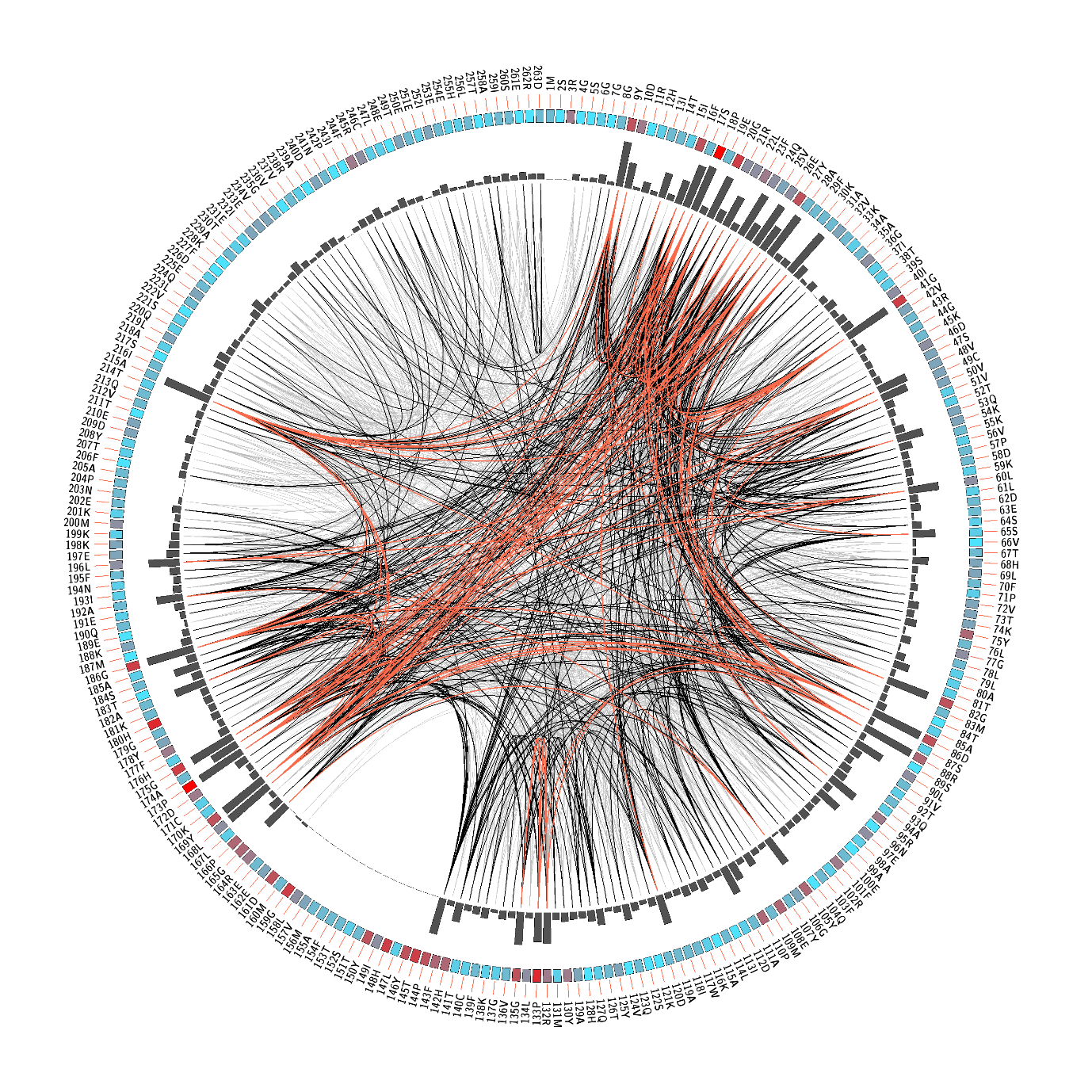
**

**Supplementary Figure 5.** Conserved and coevolved amino acid residues in α-subunit proteins in *Brassica napus*. Mutual information network, showing conserved and coevolving residues. The labels on the exterior of second circle indicate amino acid positions of α-subunit proteins of *Brassica napus.* The coloured square boxes in the second circle indicate conservation intensity (red indicates high conservation, while less conserved positions are indicated in blue). The third circle show the proximity mutual information (PMI). Curved lines in the centre connect pairs of significant MI values (> 6.5), with red lines indicating the highest MI scores (top 5%), black lines indicating midrange scores (between 70 and 95%), and grey lines indicating the lowest scores (the remaining 70%) as defined by MISTIC.


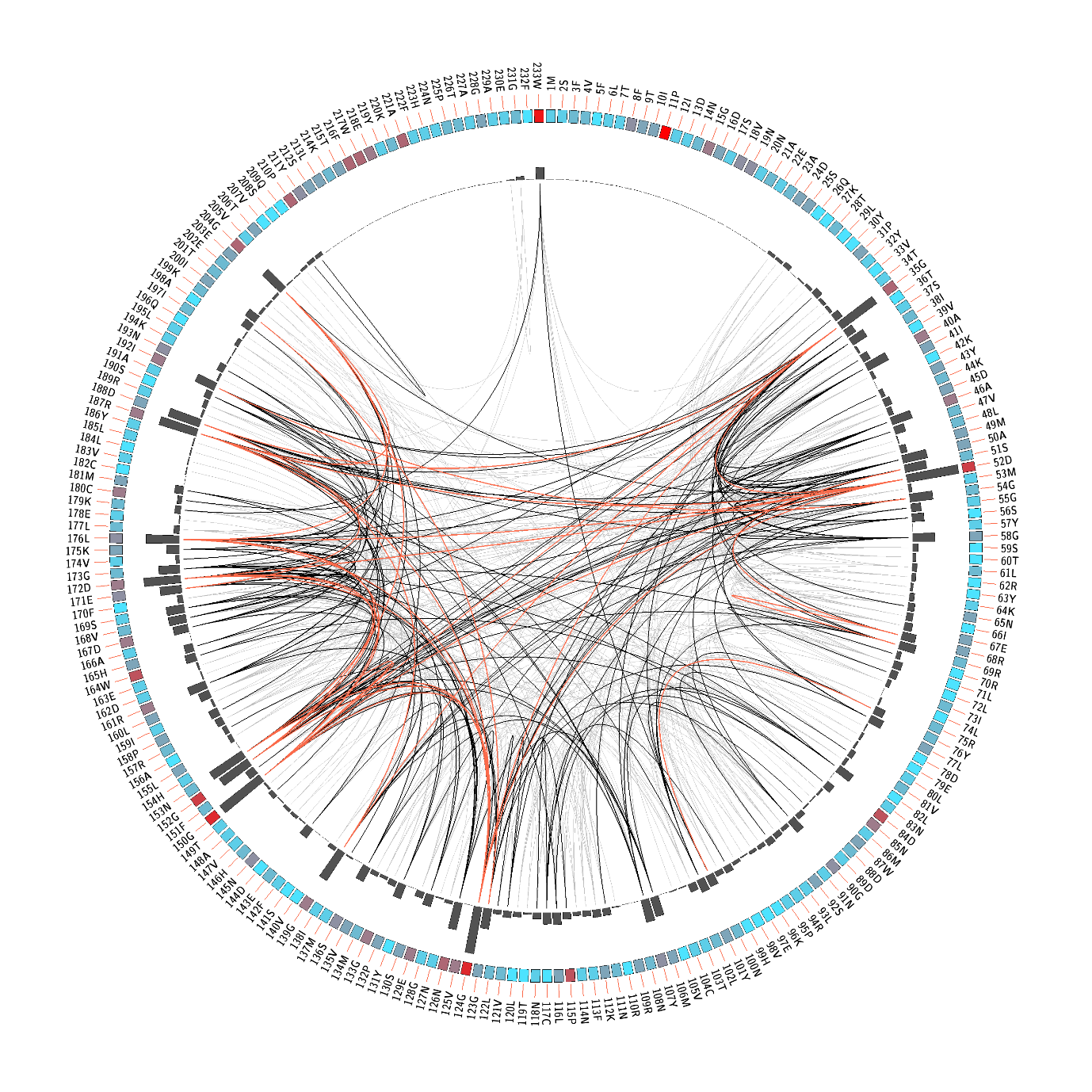


**Supplementary Figure 6.** Conserved and coevolved amino acid residues in β-subunit proteins in *Brassica napus.* Mutual information network, showing conserved and coevolving residues. The labels on the exterior of second circle indicate amino acid positions of β-subunit proteins of *Brassica napus*. The coloured square boxes in the second circle indicate conservation intensity (red indicates high conservation, while less conserved positions are in blue). The third circle show the proximity mutual information (PMI).Curved lines in the centre connect pairs of significant MI values (> 6.5), with red lines indicating the highest MI scores (top 5%), black lines indicating midrange scores (between 70 and 95%), and grey lines indicating the lowest scores (the remaining 70%) as defined by MISTIC.


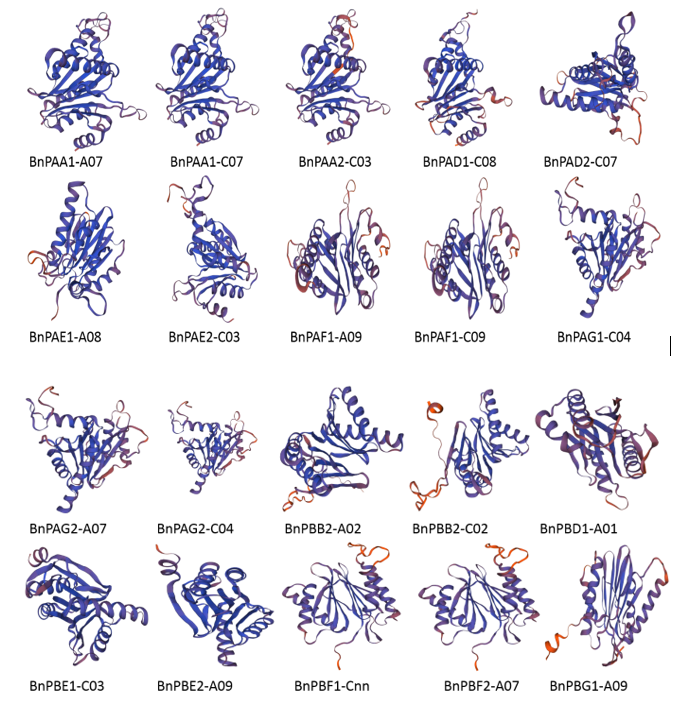


**Supplementary Figure 7.** The 3D structures of 20 proteins encoded by *BnPA* and *BnPB* genes of 20S proteasome family in *Brassica napus*. In all the 20 proteins, spirals represent helices, broad strips with arrow-head represent β-pleated sheets and thin loops represent coil.
